# Estimation of the Kinematics and Workspace of a Robot Using Artificial Neural Networks

**DOI:** 10.3390/s22218356

**Published:** 2022-10-31

**Authors:** Cătălin Boanta, Cornel Brișan

**Affiliations:** Department of Mechatronics and Machine Dynamics, Faculty of Automotive, Mechatronics and Mechanical Engineering, Technical University of Cluj-Napoca, 400114 Cluj-Napoca, Romania

**Keywords:** machine learning, neural network, feedforward, robot, kinematic analysis, workspace, artificial intelligence

## Abstract

At present, in specific and complex industrial operations, robots have to respect certain requirements and criteria as high kinematic or dynamic performance, specific dimensions of the workspace, or limitation of the dimensions of the mobile elements of the robot. In order to respect these criteria, a proper design of the robots has to be achieved, which requires years of practice and a proper knowledge and experience of a human designer. In order to assist the human designer in the process of designing the robots, several methods (including optimization methods) have been developed. The scientific problem addressed in this paper is the development of an artificial intelligence method to estimate the size of the workspace and the kinematics of a robot using a feedforward neural network. The method is applied on a parallel robot composed of a base platform, a mobile platform and six kinematic rotational-universal-spherical open loops. The numerical results show that, with proper training and topology, a feedforward neural network is able to estimate properly values of the volume of the workspace and the values of the generalized coordinates based on the pose of the end effector.

## 1. Introduction

At present, industrial robots are almost a prerequisite in industrial applications such as milling, drilling, painting, machining or pick-and-place applications, mostly due to the fact that industrial processes have become more and more complex. This is why, with the increase in complexity of the industrial operations, in order to meet the specific criteria of the several industrial processes, robots are being continuously redesigned in what regards the architecture or the control system.

The action of redesigning a robot is usually realized by human designers, who have developed several mathematical methods to improve the design and reduce the required amount of time. Most of these mathematical methods are based on optimization algorithms (for numerical optimization), but, in past years, several artificial intelligence-based methods were developed. These newly developed methods are efficient and may decrease the required computational time of the redesign process. Undoubtedly, there are some disadvantages, such as the limited capability of online implementation and the capability of neural network to identify/establish a relation between the input and the output is dependent by the quality and the size of the training data.

When it comes to the process of designing or redesigning, one important aspect is the kinematics of the robot. In addition, in recent years, several scientific papers have proposed, for the design of robots, the use of neural networks for kinematic analysis. Although these papers focus on the estimation of the generalized coordinates based on the poses of the tool center point of the robot, little work has been conducted considering the workspace as a whole as the output of a neural network based on the generalized coordinates. 

Moreover, the process of redesigning a robot is required by the applications in which the robot will be used and by the specific criteria, either imposed by the application or a human designer. In order to meet the criteria, several complex mathematical models have to be developed, which are based on highly nonlinear equations. Considering this aspect, the design of robots requires a large amount of experience from a human designer, an aspect that may be bypassed or improved with the mean of optimization methods [1,2,3,4,5,6,7]. Moreover, recently, a high amount of work was performed for the mathematical modeling of industrial robots using machine learning methods, as presented in [8,9,10]. 

In the following, several examples are presented using neural networks in order to estimate the kinematics of robots. The authors of [11] proposed the use of neural networks for the offline estimation of the kinematic analysis of a robot and an online neural network in order to control the trajectory. A multilayer feedforward neural network was trained in [12] in order to solve the kinematic analysis of a planar parallel mechanism. The proposed solution overcomes some limitations of the Newton–Rapson numerical algorithm in what regards obtaining multiple solutions for the generalized coordinates. 

Another example is [13], which proposed a supervised learning neural network in order to solve the inverse kinematic problem of serial robots, considering the assembly errors in the joints. In [14], the authors proposed adversarial neural network in order to solve the inverse kinematic and dynamic problems. 

A more complex analysis was presented in [15], in which the inverse kinematic problem of a parallel robot was approached both in analytic form and using neural networks. Three types of neural networks were used: Multilayer Perceptron (MLP), Long-Short Term Memory (LSTM) and Gated Recurrent Unit (GRU). The networks were trained and tested using a point cloud that represents the possible poses of the final effector and the corresponding generalized coordinates. The type of neural network that achieved the best accuracy was the GRU, which presented also the lowest demanded computational time. 

A similar analysis was performed in [16], where the direct kinematic problem of a three-degrees-of-freedom redundant parallel robot was resolved based on different type of machine learning methods: an MLP neural network, a Radial Basis Function (RBF) neural network and Support Vector Machines (SVM). A backpropagation neural network was used to identify a parameter of the architecture of a parallel robot based on several performance indices as the workspace, the global conditioning index and the global rigidity index. The paper presented, also, some of the disadvantages of using the neural network for the kinematic analysis of robots, such as the neural networks may not be used in an online application (and the control of robots requires real time processing), the capabilities of the neural network to identify a mathematical function between the input and output are completely dependent on the way the system was trained and the precision of the discretization (for obtaining highly accurate results, a large amount of data is required), and a slow convergence ratio.

Furthermore, ref. [17] proposed the estimation of the kinematic analysis of a parallel robot that contains six prismatic–sphere–sphere open loops by implementing several machine learning methods, such as Multiple Linear Regression, Multi-Variate Polynomial Regression, Support Vector Regression, Decision Tree Regression and Random Forest Regression.

The direct kinematic problem of an industrial robot solved using a feedforward Multilayer Perceptron was presented in [18]. A cloud of points that describes the poses of the end effector of the robot were generated using Denavit–Hartenberg matrices. A part of the points was used for training the network and the other part for testing and verification. A similar work was presented in [19], where the authors used a Deep Neural Network in order to solve the kinematics of a serial robot with five degrees of freedom. 

As presented in [20], the workspace, e.g., the geometrical locus of all the possible coordinates of the tool center point, is one of the main criteria in the design of robots. The importance of the workspace is provided by: the capability of execution of a certain task (the poses of the tool center for a certain task must lay inside the workspace), safety criteria (if a worker occupies the workspace of a robot that performs a task he or she might be injured) or factory floor or enclosure dimensioning and nesting (the environment of the robot has to allow the free movement of the tool center point). In what regards workspace estimation using neural networks, in the scientific literature, there are few published papers that treat this subject. For example, ref. [21] proposed the use of a deep neural network to estimate the workspace by the mean of subspace learning. The paper states that the computational time is lower than classical methods of workspace generation. The paper [22] presented a development of the work from [21], i.e., the generation of the full workspace of serial robots using a deep-learning framework for a given pose.

The work presented in this paper is an extended version of [23] and presents an artificial neural network-based method (a feedforward neural network) that is used to estimate the kinematics and the volume of the workspace of a robot, considering as inputs the parameters that describe the architecture of the robot and the pose of the end effector. The proposed framework is applied on a six-degrees-of-freedom parallel manipulator with rotational active joints. The novelty of the paper rests mostly on two aspects. The first aspect is the fact that the neural network estimates the values of the generalized coordinates from each active joint in particular (for each generalized coordinate, there is an output from the network), an aspect that has the advantage of and offers the possibility to apply the method for manipulators with a different number of active joints. The second one is the fact that, in the process of designing or redesigning a robot, a human designer can estimate the workspace directly, each time a parameter of the robot is changed, without the requirement of computing it with the classical workspace generation methods. 

## 2. Proposed Method

**Figure 1:**
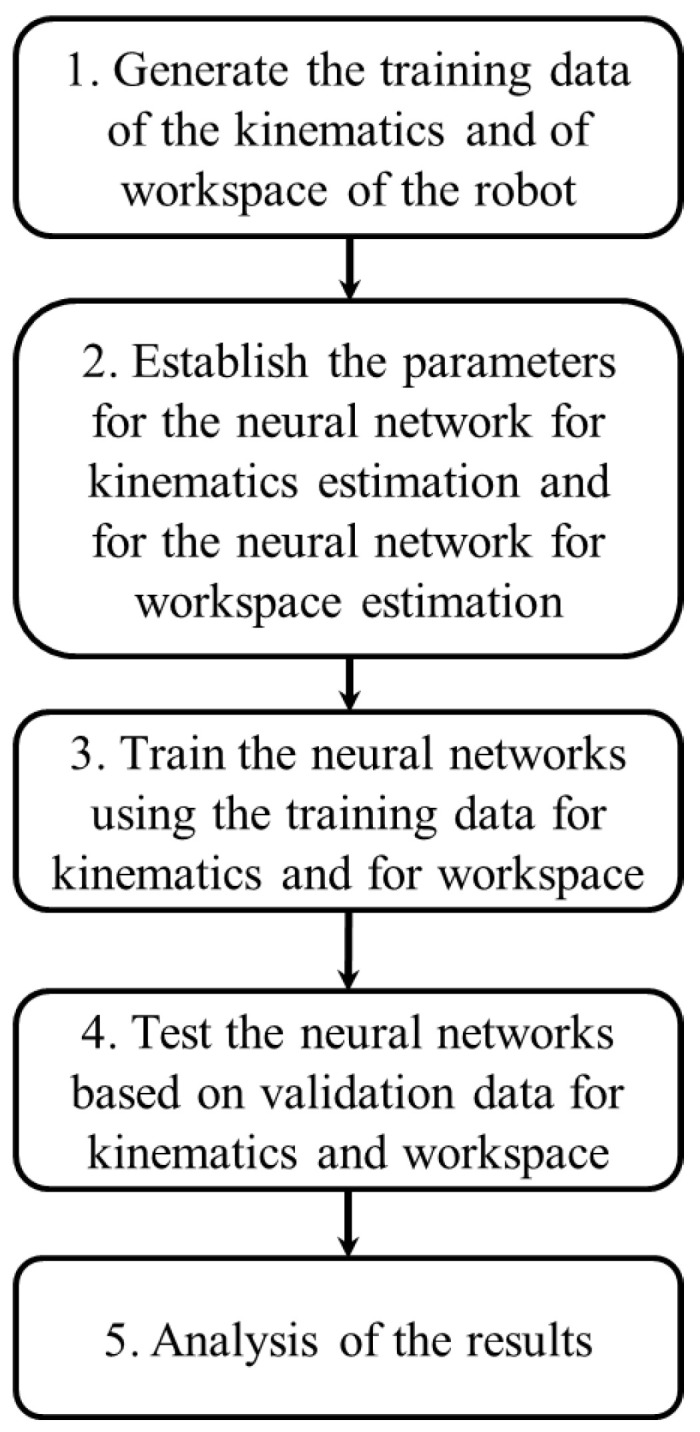
Representation of the problem formulation.

The work presented in this paper is summarized by Figure 1. In the following subsections, each step from the problem formulation is described in detail. 

### 2.1. Generate the Training Data and of the Workspace of the Robot

In the first step, the kinematic model of a robot was developed. Therefore, for a given pose of the end effector, the mathematical equations that map the values of the generalized coordinates from the active joints were developed, as in Equation (1):

(1) $$q=f\left(p,\;\varphi \right)$$

where q is a linear vector with the generalized coordinates, f represents the mathematical functions, and p and φ represent the pose of the end effector (p is the vector of the coordinates on the x, y and z axes, and φ is the vector of Euler’s angles around the x, y and z axes).

By performing the kinematics, a dataset of values for the pose of the end effector and the corresponding values for the generalized coordinates was generated. Part of this data represent the training data and the rest was kept for validation.

Next, the generation of the workspace data was based on the kinematic analysis. There are several methods proposed in the scientific literature for workspace evaluation, with the previous work of the authors on this subject being presented in [24,25]. Firstly, a 3D space (a parallelepiped) is discretized in uniformly distributed points $p_{i}$. For each $p_{i}$, the kinematic analysis is performed and, if there is a valid solution for the generalized coordinates, the point $p_{i}$ is included in the workspace. Finally, the workspace is generated as a cloud of points from all the points that generated a valid solution. 

In order to evaluate the volume of the workspace, Equation (2) is used:

(2) $$V_{WS}=v_{p_{i}}\cdot m$$

where $V_{WS}$ is the volume of the workspace, $v_{p_{i}}$ is the volume correspondent to a single point of the workspace and m is the total number of points. 

The next step was to perform multiple evaluations of the workspace using the kinematic equations, using several parameters that describe the architecture of a robot. In this way, a second dataset was created. 

For the two generated datasets (one for the kinematic analysis and one for the workspace), 80% of the data were used for training the neural networks and 20% of the data were used for validation and testing. 

### 2.2. Establish the Parameters That Describe the Neural Networks Used for Kinematics and Workspace Estimation

**Figure 2:**
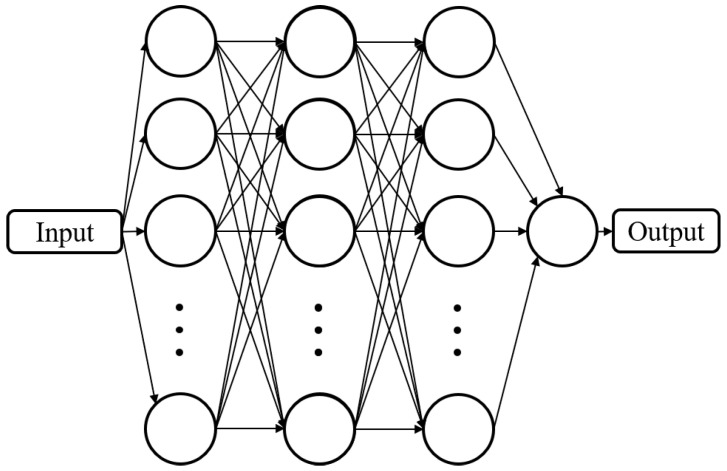
General topology of a multilayer fully connected neural network (reprinted with permission from ref. [23]. 2022, IEEE Proceedings).

The process of defining the neural network is a complex and important task and, for these applications, a proper method was not identified in the scientific literature. Both for the kinematics and workspace estimations, a multilayer, fully connected neural network was used. The network was implemented in the MATLAB environment. Figure 2 presents the general topology of the neural network. 

The parameters that describe the neural network are the number of layers, lambda λ (the regularization rate) and the type of activation function, which may be the Rectified Linear Unit (ReLU), the hyperbolic tangent tanh, the sigmoid or identity function, functions that are presented, respectively, in the following Equations (3)–(6):

(3) $$\phi \left(v\right)=\left\{\begin{array}{c} v,\;v\geq 0\\ 0,\;v<0 \end{array}\right.,$$

(4) $$\phi \left(v\right)=\tan h\left(v\right),$$

(5) $$\phi \left(v\right)=\frac{1}{1+e^{v}},$$

(6) $$\phi \left(v\right)=v.$$

In order to define these parameters, an optimization was performed in MATLAB using the Random Search Algorithm for both the networks used for the kinematics and for the workspace estimation (numerical examples of the optimization are presented in Section 4.2 and Section 5.2). 

### 2.3. Training the Neural Networks

After the establishment of the parameters that describe the networks, the neural network used for the kinematics estimation and the neural network used for the workspace estimation were trained based on the two datasets created in the first step. As presented, a fraction of the two datasets was kept for validation purposes. 

In this step, another important aspect is the convergence criterion. This may be the maximal number of function evaluations, a specific minimal value of the mean squared error (MSE) or the confirmation of the performance of the trained network based on the data reserved for validation. This criterion is imposed by the user that defines the network but may be dependent on the application the neural network is used for.

### 2.4. Tesing the Trained Neural Network Us

After the training of the neural networks from the third step, the performance of the networks was evaluated using the validation datasets (using the 20% fraction reserved in the first step from each dataset).

The outputs of the two neural networks, i.e., the estimated values of the generalized coordinates and the volume of the workspaces, were compared with the numerically evaluated ones. In order to quantify the error, two mean squared errors were computed, one for the kinematics and one for the volume of the workspace, as presented in the following equations:

(7) $$MSE_{Kinematics}=\frac{1}{t}\overset{t}{\underset{i=1}{\sum }}{\left(q_{i}-{\hat{q}}_{i}\right)}^{2}$$

(8) $$MSE_{Workspace}=\frac{1}{p}\overset{p}{\underset{i=1}{\sum }}{\left(V_{WS_{i}}-{\hat{V}}_{WS_{i}}\right)}^{2},$$

where $q_{i}$ is the ith vector with generalized coordinates evaluated numerically in the first step, ${\hat{q}}_{i}$ is the vector with estimated values of the generalized coordinates corresponding to the same input as for the numerically evaluated one, t is the total number of test cases for kinematics, $V_{WS_{i}}$ represents the numerically evaluated volume of the *i*th workspace from the first step, ${\hat{V}}_{WS_{i}}$ is the volume of the workspace that was estimated by the neural network, corresponding to the same parameters of the robot, and p is the total number of test cases for the workspace.

### 2.5. Analysis of the Performance of the Neural Networks

The last step corresponds to an in-depth analysis of the performance of the neural networks. The estimated values (both for the kinematics and for the volume of the workspace) were compared in terms of relative difference with the numerically evaluated ones (from the validation dataset). Moreover, the convergence of the neural networks, the training history and the error histograms were presented for each neural network. 

## 3. Prerequisite—Kinematics and Workspace Analysis of a Parallel Robot

In order to validate the proposed method and to generate the training and test data for the neural network, the kinematics and the workspace of a robot were developed in analytical form. In this paper, a six-degrees-of-freedom parallel robot was used, composed of a fixed platform, a mobile platform and six identical kinematic open loops that interconnect the platforms. These open loops are composed of two mobile elements, a rotational joint (R), a universal joint (U) and a spherical joint (S). 

On the one hand, the reason for applying the methodology on a parallel robot is that, in the case of parallel robots, one disadvantage is the reduced size of the workspace in comparison with the dimensions of the elements of the robots. Moreover, in the case of the direct kinematics of a parallel robot, there is a closed-form analytical solution for computing the poses of the tool center point from the values of the generalized coordinates.

On the other hand, the methodology presented in Section 2 was developed regardless of the type of the robot to which it is applied, so it may be applied to other types of robots (e.g., serial or parallel robots). 

**Figure 3:**
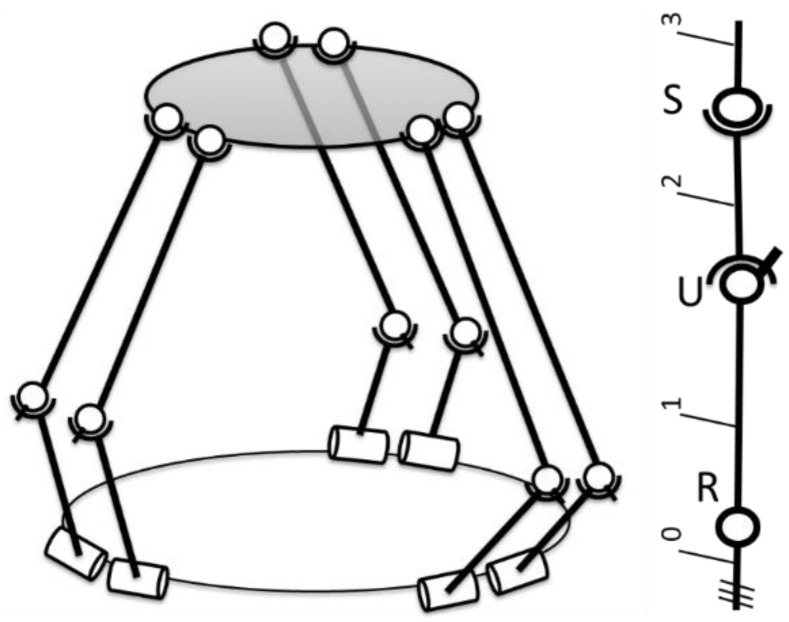
Parallel robot composed of 6 identical RUS open loops (adapted with permission from ref. [23]. 2022, IEEE Proceedings).

**Figure 4:**
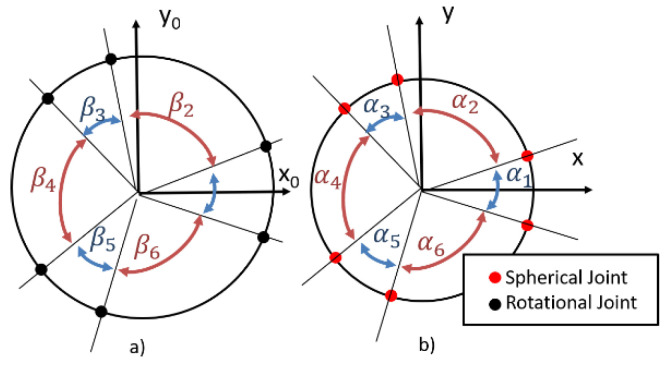
The fixed (**a**) and mobile (**b**) platforms of the parallel robot (adapted with permission from ref. [23]. 2022, IEEE Proceedings).

The architecture of the robot is presented in Figure 3 and the top view of the platforms is presented in Figure 4.

The parameters in Figure 3 and Figure 4 represent:

- $l_{1}$ and $l_{2}$ are the lengths of the elements of the RUS kinematic open loops that interconnect the platforms;
- R and r are the radii of the two platforms (fixed and mobile);
- $\beta_{i}$ and $\alpha_{i}$ are the angles that describe the positioning of the rotational joints on the fixed platform and of the spherical joints on the mobile platform, respectively (where i=1…6).

### 3.1. Kinematic Analysis

**Figure 5:**
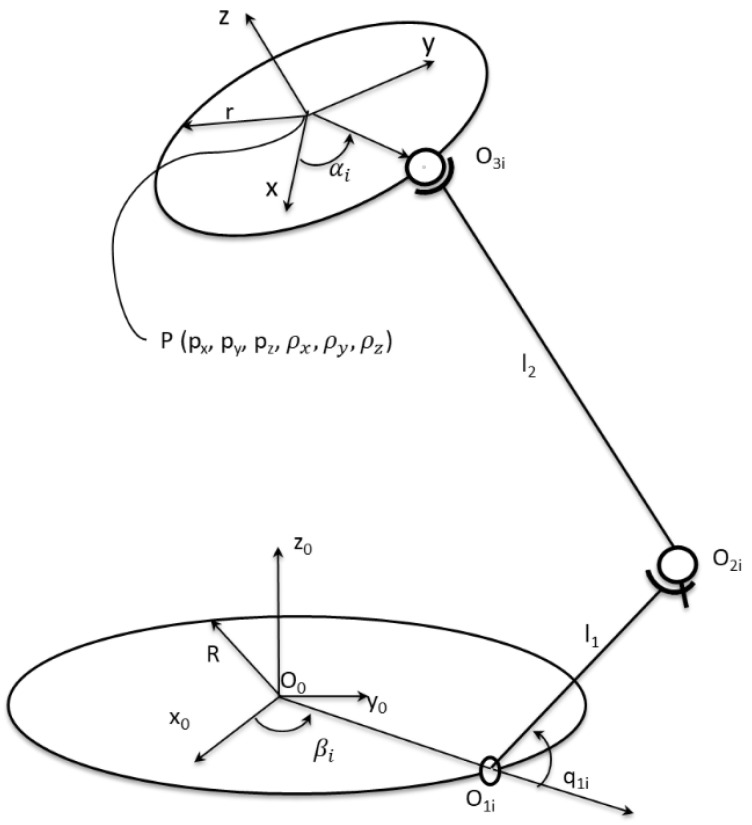
Parallel robot (only one RUS open loop is represented).

In order to perform the kinematic analysis, Figure 5 is considered. 

The notations from Figure 5 (other than ones presented in the previous two figures) represent (for each notation, i=1…6):

- $O_{0}x_{0}y_{0}z_{0}$ is the absolute coordinate system attached to the fixed platform;
- $P_{xyz}$ is the relative coordinate system attached to the mobile platform. The coordinates of the position vector p of the point P with respect to the absolute frame $O_{0}x_{0}y_{0}z_{0}$ are $\left(p_{x},\;p_{y},\;p_{z}\right)$;
- The angles $\rho_{x},\rho_{y},\rho_{z}$ are the Euler’s rotation angles of the system $P_{xyz}$ around $x_{0}$, $y_{0}$ and $z_{0}$ of the absolute coordinate system;
- $q_{1i}$ is the active angle in the rotational joint i;
- $O_{1i}$ is the center of the rotational joint. The coordinates of the position vector $o_{1i}$ of the point $O_{1i}\;$ expressed in the absolute coordinate system are $\left(o_{1ix},\;o_{1iy},\;o_{1iz}\right)$;
- $O_{2i}$ is the center of the universal joint. The coordinates of the position vector $o_{2i}$ of the point $O_{2i}$ expressed in the absolute coordinate system are $\left(o_{2ix},\;o_{2iy},\;o_{2iz}\right)$;
- $O_{3i}$ is the center of the universal joint. The coordinates of the position vector $o_{3i}$ of the point $O_{3i}$ expressed in the absolute coordinate system are $\left(o_{3ix},\;o_{3iy},\;o_{3iz}\right)$;

The kinematic analysis was solved for one open loop, in a similar manner as presented in [26]. Applying the same methodology for each open loop, the inverse kinematic analysis was solved for entire robot. Firstly, one has to evaluate the analytic expression of the active angle $q_{1i}$. The geometrical parameters of the mobile platform that are known are: $R,\;r,\;l_{1},\;d,\;l_{2},\;\beta_{i},\;\alpha_{i}.\;$ Writing the equation of the length of the second element, $l_{2}\;$ the following equation was obtained (for each equation, the value of i=1…6):

(9) $${\left(o_{3ix}-o_{2ix}\right)}^{2}+{\left(o_{3iy}-o_{2iy}\right)}^{2}+{\left(o_{3iz}-o_{2iz}\right)}^{2}=l_{2}^{2}.$$

The coordinates of the point $O_{3i}$ were evaluated from the pose of Pxyz towards $O_{0}x_{0}y_{0}z_{0}$:

(10) $${\left[o_{3ix},o_{3iy},o_{3iz}\right]}^{T}={\left[p_{x},p_{y},p_{z}\right]}^{T}+R_{zyx}\cdot r\cdot {\left[\cos\left(\propto_{i}\right),\sin\left(\propto_{i}\right),0\right]}^{T}.$$

The term $R_{zyx}$ is the rotation matrix of Pxyz towards $O_{0}x_{0}y_{0}z_{0}$, evaluated with:

(11) $$R_{zyx}=R_{z}\left(\rho_{z}\right)\cdot R_{y}\left(\rho_{y}\right)\cdot R_{x}\cdot \left(\rho_{x}\right).$$

The coordinates $\left(o_{2ix},\;o_{2iy},\;o_{2iz}\right)$ of the points $O_{2i}$ were evaluated with the equation:

(12) $${\left[o_{2ix},o_{2iy},o_{2iz}\right]}^{T}={\left[o_{1ix},\;o_{1iy},\;o_{1iz}\right]}^{T}+\;l_{1i}\cdot {\left[\cos\beta_{i}\cdot \cos q_{1i},\sin\beta_{i}\cdot \;\cos q_{1i},\sin q_{1i}\right]}^{T}.$$

The coordinates $\left(o_{1ix},o_{1iy},o_{1iz}\right)$ of the point $O_{1i}\;$ are:

(13) $${\left[o_{1ix},o_{1iy},o_{1iz}\right]}^{T}=r\cdot {\left[\cos\beta_{i},\sin\beta_{i},0\right]}^{T}.$$

By replacing Equations (10)–(13) into Equation (9), we obtained:

(14) $$\cos q_{1i}\cdot D_{i}+\sin q_{1i}\cdot E_{i}=F_{i},$$

where $D_{i}$, $E_{i}$ and $F_{i}$ are evaluated as follows:

(15) $$D_{i}=\cos\beta_{i}\left(o_{3ix}-o_{1ix}\right)+\sin\beta_{i}\left(o_{3iy}-o_{1iy}\right),$$

(16) $$E_{i}=o_{3iz}-o_{1iz}$$

(17) $$F_{i}=\frac{\left[l_{1}^{2}-l_{2}^{2}+{\left(o_{3ix}-o_{1ix}\right)}^{2}+{\left(o_{3iy}-o_{1iy}\right)}^{2}+{\left(o_{3iz}-o_{1iz}\right)}^{2}\right]}{\left(2\;\cdot l_{1}\right)}.$$

The analytic expression of the active angle $q_{1i}$ is expressed by:

(18) $$\sin\left(q_{1i}\right)=\frac{\left(E_{i}\cdot F_{i}-K_{i}\cdot D_{i}\cdot \sqrt{\left(D_{i}{}^{2}+E_{i}{}^{2}-F_{i}{}^{2}\right)}\right)}{\left(D_{i}^{2}+E_{i}^{2}\right)},$$

(19) $$\cos\left(q_{1i}\right)=\frac{\left(D_{i}\cdot F_{i}+K_{i}\cdot E_{i}\cdot \sqrt{\left(D_{i}{}^{2}+E_{i}{}^{2}-F_{i}{}^{2}\right)}\right)}{\left(D_{i}^{2}+E_{i}^{2}\right)}.$$

The term $K_{i}$ from Equations (18) and (19) has the value either 1 or −1, depending on the joint configuration (branch index). Each angle $q_{1i}$ has two valid solutions in the inverse kinematic problem, depending on the term $K_{i}$. Only one of them was considered in the following equations. The final solution for the active angles was evaluated with the atan2 function:

(20) $$q_{1i}=atan2\left(\sin\left(q_{1i}\right),\cos\left(q_{1i}\right)\right).$$

### 3.2. Workspace Analysis

In general, the workspace of a robot is defined by the space occupied by the end effector considering all the possible positions and orientation of its end effector. In the following, the zero-orientation workspace was developed for the six-degrees-of-freedom parallel robot analyzed in this paper. There several constraints that affect the workspace, as presented in [20] as:

1. Kinematic/geometrical constraints represent the solutions of the active angles from the inverse kinematics from Section 3.2:
  (21) $$q_{i}=f\left(p_{x},\;p_{y},\;p_{z},\varphi_{x},\;\varphi_{y},\;\varphi_{z}\;\right).$$
2. Mechanical constraints are the physical limitations in the joints:
  (22) $$q_{i\;min}\leq q_{i}\leq q_{i\;max}.$$
3. Limitations regarding the minimal distance between elements ($e_{k}$ and $e_{l}$ being two random elements):
  (23) $$dist\left(e_{k},e_{l}\right)>dist_{min}\;$$

**Figure 6:**
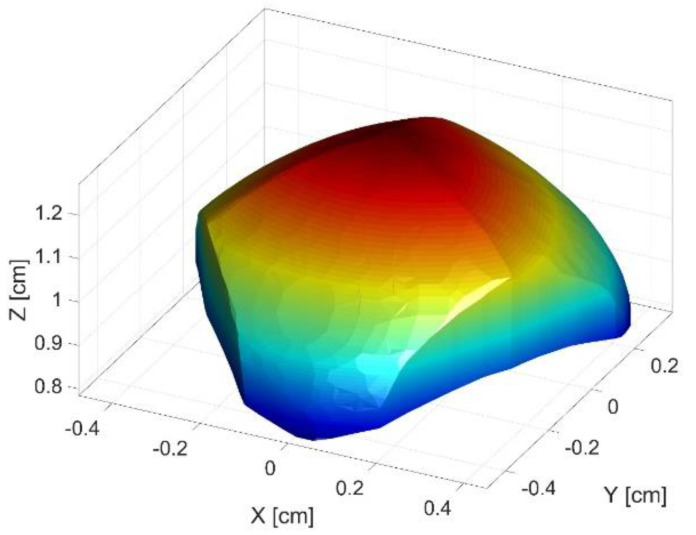
Workspace of the parallel robot.

Firstly, a cube is discretized in points for which the inverse kinematic problem is evaluated. If there is a geometric solution of the kinematics, it is evaluated if the point lies within the singular configurations and if other mechanical constraints are respected. If all the requirements are true, the analyzed point lies within the workspace. By combining all the valid points from the discretized cube, the workspace of the robot is generated. The workspace of the parallel robot is presented in Figure 6.

## 4. Results—Estimation of the Kinematics Using a Neural Network

In this section, the methodology presented in Section 2 was applied for the inverse kinematics of the six-degrees-of-freedom parallel robot, i.e., a feedforward neural network was trained to estimate the values of the generalized coordinates based on the pose of the end effector. 

### 4.1. Generation of the Training and Test Dataset

The desired output of the neural network is the generalized coordinates given the pose of the end effector. The training data have to be generated, i.e., a dataset of poses of the end effector are randomly generated (which is the input in the neural network) and the corresponding generalized coordinates are evaluated using the inverse kinematics algorithm presented in Section 3 (the generalized coordinates are the output of the neural network). For each generalized coordinate, the neural network was trained individually meaning that there were six models trained, for each generalized coordinate.

The pose of the end effector is given by the coordinates of the tool center point $\left(p_{x},\;p_{y},\;p_{z}\right)$ on the x, y and z axes and the angles of rotations around each axis $\left(\rho_{x},\rho_{y},\rho_{z}\right)$. In order to generate the training data, a number of 10,000 random values were considered for each coordinate and angle. The random values were generated between the following minimal and maximal limits for each parameter, as presented in Equation (24):

(24) $$-0.3<p_{x}<0.3\;\left[m\right],\;-0.3<p_{y}<0.3\;\left[m\right],\;0.8<p_{z}<1.2\left[m\right],\;-\frac{\pi }{6}<\rho_{x}<\frac{\pi }{6},\;-\frac{\pi }{6}<\rho_{y}<\frac{\pi }{6},\;-\frac{\pi }{12}<\rho_{z}<\frac{\pi }{12}.$$

The random values were combined in 10,000 input random combinations. For each combination, the correspondent generalized coordinates of the robot were evaluated using the inverse kinematics analysis. In this way, the dataset required for training the neural network was generated. A fraction of 80% of this dataset was reserved for the training of the neural network and the remaining 20% was reserved for validation. 

### 4.2. Define the Parameters of the Neural Network for Kinematic Estimation

The type of network that was used to evaluate the volume of a workspace based on the input parameters was a feedforward, fully connected neural network implemented in the MATLAB environment. The parameters that define the topology of the neural network have a strong influence upon the numerical results. This is why, in order to increase the efficiency, the parameters of the network were established based on an optimization. 

The optimization was run using a random search algorithm from the MATLAB environment. The optimization was run for 1000 iterations and, on each iteration, the neural network was trained based on the training data already prepared. The objective function of the optimization was the mean squared error after training. On each iteration, the neural network was trained up to the point in which the number of epochs for training reached 1000. The resulting parameters that provided the best neural network are presented in Equations (25)–(28):

(25) $$No_{layers}=2,\;$$

(26) $$Size_{layers}=\left[18,13\right]$$

(27) $$\lambda =7.943\times 10^{-8},\;\;$$

(28) $$Act_{fcn}=tanh.$$

In Equations (25)–(28), the parameters that were optimized are: $No_{layers}$—the number of intermediate layers (without the input and output layers), $Size_{layers}$—a vector that contains the size (the number of neurons) of each intermediate layer, λ—the regularization rate and $Act_{fcn}$—the type activation function.

### 4.3. Numerical Results

The feedforward neural network was trained in the MATLAB environment using 80% of the training data. The equipment that was used was a PC with an Intel I7-4770k processor and 64 GB of RAM. 

As presented before, six trained models were generated, using the same neural network parameters each time. Therefore, each model estimated one generalized coordinate. In each case, the condition of convergence of the neural network was reaching 1 million epochs or an MSE of $1\times 10^{-7}$. 

#### 4.3.1. Results for the First Generalized Coordinate $q_{1}$

**Figure 7:**
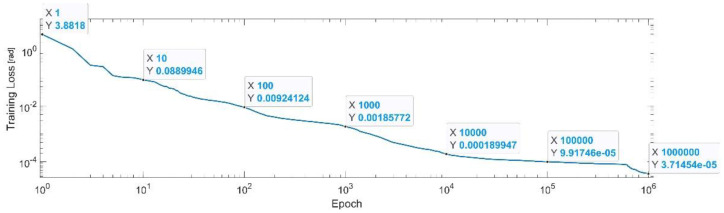
Convergence of the neural network for the first generalized coordinate $q_{1}$ (log scale).

Figure 7 presents the convergence of the neural network on a logarithm scale. From the 1st epoch to the 1000th epoch, the mean squared error (MSE) reached from 3.88 to 1.8 × 10^−3^. In the 1 millionth epoch, the MSE reached its lowest value, as in Equation (29).


(29) $$MSE\_Best\_q_{1}=3.71\times 10^{-5}.$$

**Figure 8:**
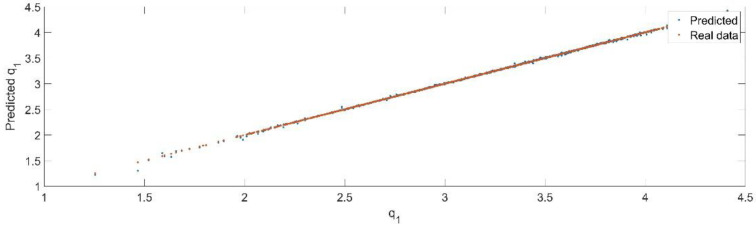
The comparison between the real data (the real value of $q_{1}$) and the predicted data (the predicted value of $q_{1}$).

Using the trained model, the values of the first generalized coordinate were estimated, using the fraction of 20% from the dataset reserved for validation. Figure 8 presents the real values of the first generalized coordinate in comparison with the estimated ones from the neural network.

**Figure 9:**
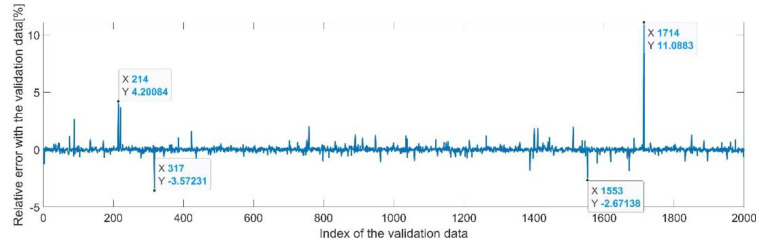
Relative errors between the target values and the predicted values for $q_{1}$.

**Figure 10:**
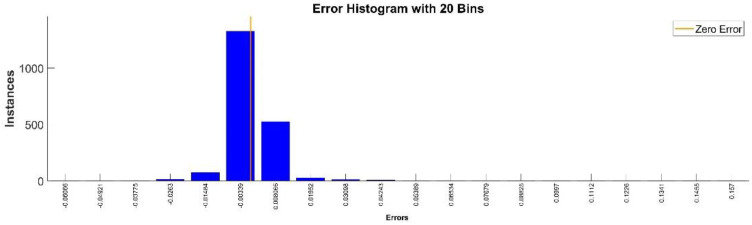
Error histogram between the target values and the estimated values for $q_{1}$.

Figure 9 presents the relative error between the real validation data and the predicted data and the model and Figure 10 presents the error histogram between the target and predicted values. 

By analyzing Figure 9 and Figure 10, the maximal error between the target and prediction was 11.08%. Nevertheless, among the 2000 target values, only for 17, the relative error was higher than 1%. The mean error between the target and prediction for the first generalized coordinate was 0.0103%. 

#### 4.3.2. Results for the First Generalized Coordinate $q_{2}$

**Figure 11:**
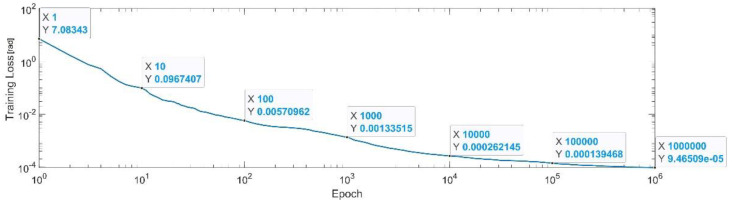
Convergence of the neural network for the first generalized coordinate $q_{2}$ (log scale).

Figure 11 presents the convergence of the neural network on a logarithm scale. From the 1st epoch to the 1000th epoch, the mean squared error reached from 7.08 to 1.3 × 10^−3^. In the 1 millionth epoch, the MSE reached its lowest value, as in Equation (30).


(30) $$MSE\_Best\_q_{2}=9.46\times 10^{-5}.$$

**Figure 12:**
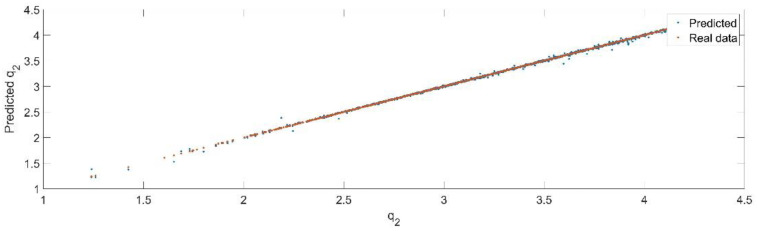
The comparison between the real data (the real value of $q_{2}$) and the predicted data (the predicted value of $q_{2}$).

Using the trained model, the values of the first generalized coordinate were estimated, using the fraction of 20% from the dataset reserved for validation. Figure 12 presents the real values of the first generalized coordinate in comparison with the estimated ones from the neural network.

**Figure 13:**
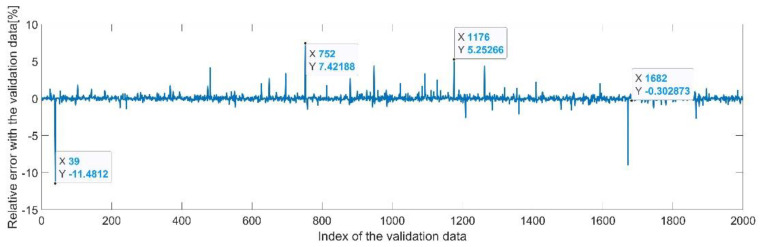
Relative errors between the target values and the predicted values for $q_{2}$.

**Figure 14:**
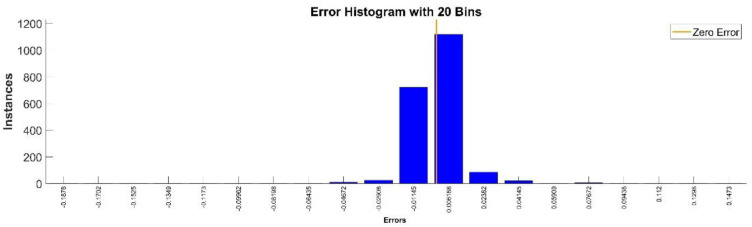
Error histogram between the target values and the estimated values for $q_{2}$.

Figure 13 presents the relative error between the real validation data and the predicted data and the model and Figure 14 presents the error histogram between the target and predicted values. 

By analyzing Figure 13 and Figure 14, the maximal error between the target and prediction was −11.48%. Nevertheless, among the 2000 target values, only for 38, the relative error was higher than 1%. The mean error between the target and prediction for the second generalized coordinate was 0.104%. 

#### 4.3.3. Results for the First Generalized Coordinate $q_{3}$

**Figure 15:**
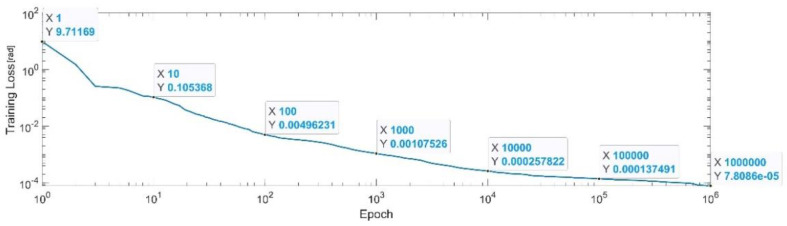
Convergence of the neural network for the first generalized coordinate $q_{3}$ (log scale).

Figure 15 presents the convergence of the neural network on a logarithm scale. From the 1st epoch to the 1000th epoch, the mean squared error reached from 9.71 to 1.07 × 10^−3^. In the 1 millionth epoch, the MSE reached its lowest value, as in Equation (31).


(31) $$MSE\_Best\_q_{3}=7.8\times 10^{-5}.$$

**Figure 16:**
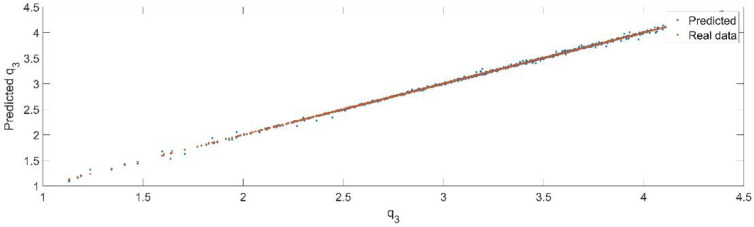
The comparison between the real data (the real value of $q_{3}$) and the predicted data (the predicted value of $q_{3}$).

Using the trained model, the values of the first generalized coordinate were estimated, using the fraction of 20% from the dataset reserved for validation. Figure 16 presents the real values of the first generalized coordinate in comparison with the estimated ones from the neural network.

**Figure 17:**
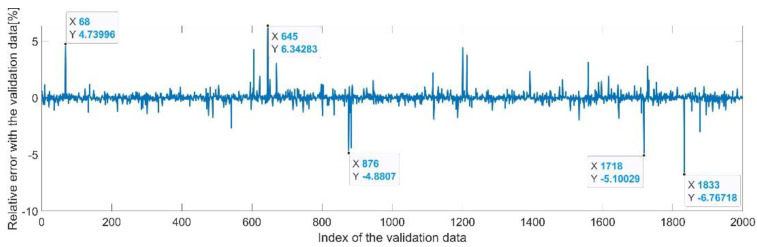
Relative errors between the target values and the predicted values for $q_{3}$.

**Figure 18:**
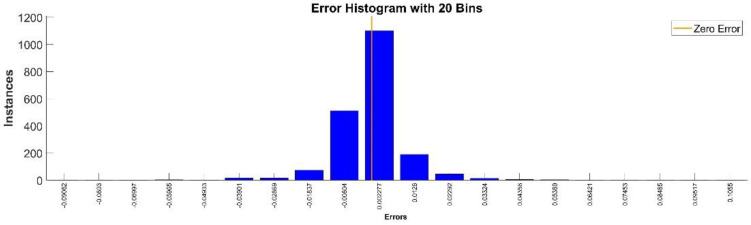
Error histogram between the target values and the estimated values for $q_{3}$.

Figure 17 presents the relative error between the real validation data and the predicted data and the model and Figure 18 presents the error histogram between the target and predicted values. 

By analyzing Figure 16 and Figure 18, the maximal error between the target and prediction was −6.76%. Nevertheless, among the 2000 target values, only for 29, the relative error was higher than 1%. The mean error between the target and prediction for the third generalized coordinate was 0.042%. 

#### 4.3.4. Results for the First Generalized Coordinate $q_{4}$

**Figure 19:**
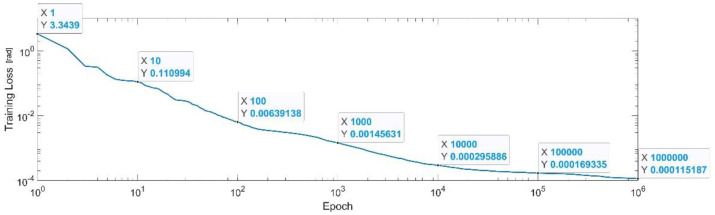
Convergence of the neural network for the first generalized coordinate $q_{4}$ (log scale).

Figure 19 presents the convergence of the neural network on a logarithm scale. From the 1st epoch to the 1000th epoch, the mean squared error reached from 3.34 to 1.4 × 10^−3^, In the 1 millionth epoch, the MSE reached its lowest value, as in Equation (32).


(32) $$MSE\_Best\_q_{4}=1.14\times 10^{-4}.$$

**Figure 20:**
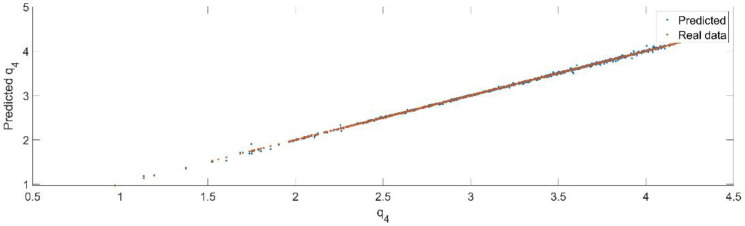
The comparison between the real data (the real value of $q_{4}$) and the predicted data (the predicted value of $q_{4}$).

Using the trained model, the values of the first generalized coordinate were estimated, using the fraction of 20% from the dataset reserved for validation. Figure 20 presents the real values of the first generalized coordinate in comparison with the estimated ones from the neural network.

**Figure 21:**
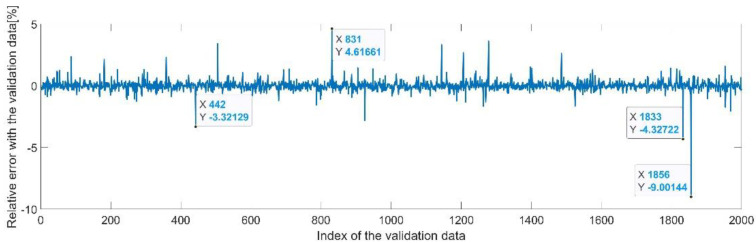
Relative errors between the target values and the predicted values for $q_{4}$.

**Figure 22:**
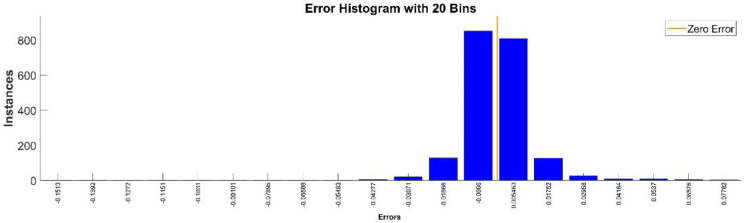
Error histogram between the target values and the estimated values for $q_{4}$.

Figure 21 presents the relative error between the real validation data and the predicted data and the model and Figure 22 presents the error histogram between the target and predicted values. 

By analyzing Figure 21 and Figure 22, the maximal error between the target and prediction was around −9%. Nevertheless, among the 2000 target values, only for 33, the relative error was higher than 1%. The mean error between the target and prediction for the fourth generalized coordinate was 0.038%. 

#### 4.3.5. Results for the First Generalized Coordinate $q_{5}$

**Figure 23:**
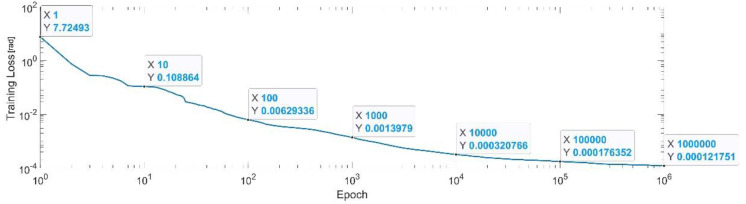
Convergence of the neural network for the first generalized coordinate $q_{5}$ (log scale).

Figure 23 presents the convergence of the neural network on a logarithm scale. From the 1st epoch to the 1000th epoch, the mean squared error reached from 7.72 to 1.3 × 10^−3^. In the 1 millionth epoch, the MSE reached its lowest value, as in Equation (33).


(33) $$MSE\_Best\_q_{5}=1.2\times 10^{-4}.$$

**Figure 24:**
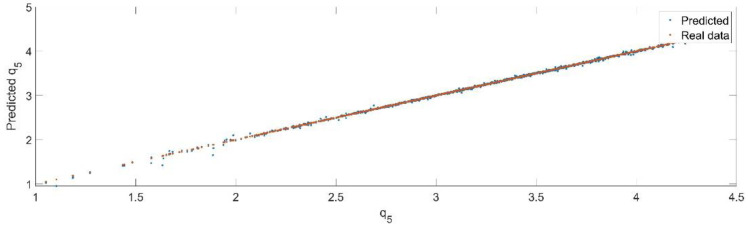
The comparison between the real data (the real value of $q_{5}$) and the predicted data (the predicted value of $q_{5}$).

Using the trained model, the values of the first generalized coordinate were estimated, using the fraction of 20% from the dataset reserved for validation. Figure 24 presents the real values of the first generalized coordinate in comparison with the estimated ones from the neural network.

**Figure 25:**
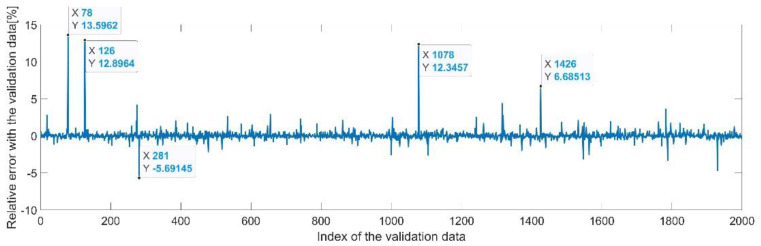
Relative errors between the target values and the predicted values for $q_{5}$.

**Figure 26:**
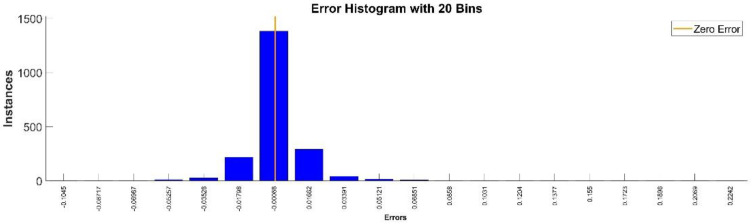
Error histogram between the target values and the estimated values for $q_{5}$.

Figure 25 presents the relative error between the real validation data and the predicted data and the model and Figure 26 presents the error histogram between the target and predicted values. 

By analyzing Figure 25 and Figure 26, the maximal error between the target and prediction was 13.59%. Nevertheless, among the 2000 target values, only for 38, the relative error was higher than 1%. The mean error between the target and prediction for the fifth generalized coordinate was 0.0309%. 

#### 4.3.6. Results for the First Generalized Coordinate $q_{6}$

**Figure 27:**
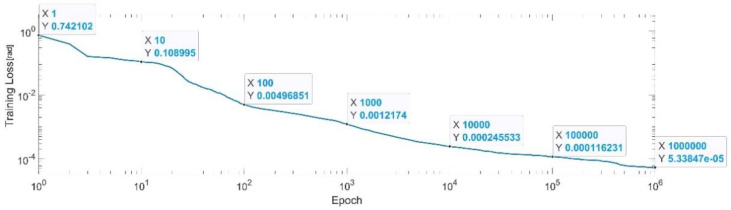
Convergence of the neural network for the first generalized coordinate $q_{6}$ (log scale).

Figure 27 presents the convergence of the neural network on a logarithm scale. From the 1st epoch to the 1000th epoch, the mean squared error reached from 0.72 to 1.2 × 10^−3^, In the 1 millionth epoch, the MSE reached its lowest value, as in Equation (34).


(34) $$MSE\_Best\_q_{6}=5.37\times 10^{-5}.$$

**Figure 28:**
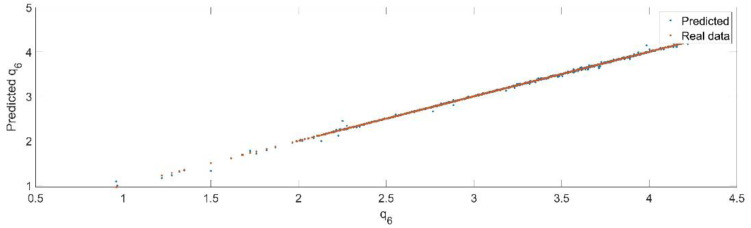
The comparison between the real data (the real value of $q_{6}$) and the predicted data (the predicted value of $q_{6}$).

Using the trained model, the values of the first generalized coordinate were estimated, using the fraction of 20% from the dataset reserved for validation. Figure 28 presents the real values of the first generalized coordinate in comparison with the estimated ones from the neural network.

**Figure 29:**
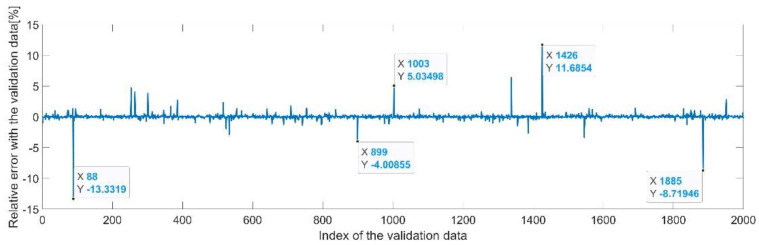
Relative errors between the target values and the predicted values for $q_{6}$.

**Figure 30:**
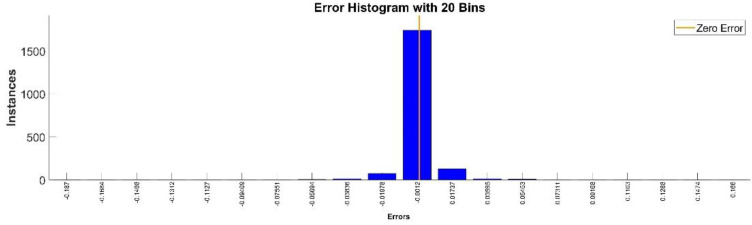
Error histogram between the target values and the estimated values for $q_{6}$.

Figure 29 presents the relative error between the real validation data and the predicted data and the model and Figure 30 presents the error histogram between the target and predicted values. 

By analyzing Figure 29 and Figure 30, the maximal error between the target and prediction was −13.3%. Nevertheless, among the 2000 target values, only for 27, the relative error was higher than 1%. The mean error between the target and prediction for the sixth generalized coordinate was 0.0058%. 

#### 4.3.7. Comparison of the Results for the Generalized Coordinates 

The numerical results for all generalized coordinates are similar in what regards the magnitude of each MSE after 1 million epochs, maximal errors and the number of values with errors higher than 1%. 

In what regards the MSE, it reached similar degrees of magnitude for each generalized coordinate; the highest value was in the case of $q_{4}$, where the MSE was $12.1\times 10^{-5}$, and the lowest was in the case of $q_{1}$, where the MSE was $3.7\times 10^{-5}$. 

The highest errors of estimation of the generalized coordinates were around 11–13% for $q_{1}$, $q_{2}$, $q_{5}$ and $q_{6}$ and around 6–9%  for $q_{3}$ and $q_{4}$, which may seem high in absolute value. Still, the maximal number of relative errors higher than 1% is 38, among 2000 validation cases for each generalized coordinate, which leads to a ratio of 1.9%. Moreover, considering the fact that the values of the mean relative errors for the estimation of each generalized coordinate were below 0.104% (correspondent for the case of $q_{2}$), it can be concluded that the estimation of the values of the generalized coordinate using the proposed methodology has a good level of accuracy.

## 5. Results—Estimation of the Workspace Using a Neural Network

In this section, we applied the methodology presented in Section 2 in order to estimate the volume of the workspace of the parallel robot presented in Section 3. 

### 5.1. Generation of the Training and Test Dataset

The desired output of the neural network is the volume of the workspace given the parameters that describe the architecture of the robot. Therefore, in this step, the training data has to be generated, i.e., a dataset of workspaces is computed (which is the output of the neural network) based on the parameters that define the architecture of the robot (which are the input of the neural network). 

By evaluating all the parameters that define the architecture of the robot, the ones that were considered as most influent upon the size of the workspace were: $l_{1}$ and $l_{2}$, the lengths of the mobile elements of the RUS kinematic open loop, R and r, the radii of the fixed and mobile platforms, respectively, and $\beta_{i}$ and $\alpha_{i}$, the angles of positioning of the rotational joints on the fixed platforms and of the spherical joints on the mobile platform, respectively. Since the angles correspondent to an odd value of the index i are equal to each other and the angles correspondent to an odd value of the index i are equal to each other, in order to reduce the number of parameters, the ratios between two consecutive angles from the fixed and from the mobile platforms were introduced, notated with $ratio_{d}$ and $ratio_{u}$, expressed in Equations (35) and (36) as:

(35) $$ratio_{d}=\frac{\beta_{1}}{\;\beta_{2}}=\frac{\beta_{3}}{\;\beta_{4}}=\frac{\beta_{5}}{\;\beta_{6}},$$

(36) $$ratio_{u}=\frac{\alpha_{1}}{\;\alpha_{2}}=\frac{\alpha_{3}}{\;\alpha_{4}}=\frac{\alpha_{5}}{\;\alpha_{6}}.$$

Therefore, the input parameters for the neural network for estimation of the volume of the workspace were $l_{1},\;l_{2},\;R,\;r,\;ratio_{d}$ and $ratio_{u}$. In order to generate the training data, for each of the parameters, a number of 10,000 random values were considered. The random values were generated between the minimal and maximal limits for each parameter:

(37) $$0.1<l_{1}<0.3\;\left[m\right],\;0.2<l_{2}<0.9\;\left[m\right],\;0.1<r<0.3\left[m\right],\;0.2<R<0.5\;\left[m\right],\;0.5<ratio_{d}<2,\;0.5<ratio_{u}<2.\;$$

With these random variables, a number of 10,000 of robot workspaces were evaluated, corresponding to 10,000 input parameters. A fraction of 80% of this dataset was reserved for training of the neural network and the rest of 20% was reserved for validation. 

### 5.2. Define of the Parameters of the Neural Network

Similar to Section 4, the type of network that was used to evaluate the volume of a workspace based on the input parameters was a feedforward, fully connected neural network implemented in the MATLAB environment. In order to establish the parameters of the neural network, another optimization was run using a random search algorithm from the MATLAB environment. The optimization was run for 1000 iterations and, on each iteration, the neural network was trained based on the training data already prepared. On each iteration, the neural network was trained up to the point in which the number of epochs reached 1000. The following parameters were obtained for the neural network:

(38) $$No_{layers}=2,\;$$

(39) $$Size_{layers}=\left[30,11\right],$$

(40) $$\lambda =1.6331\times 10^{-8},\;\;$$

(41) $$Act_{fcn}=tanh.$$

In Equations (38)–(41), the parameters represent the same as the ones from Equations (25)–(28).

### 5.3. Numerical Results for the the Workspace

**Figure 31:**
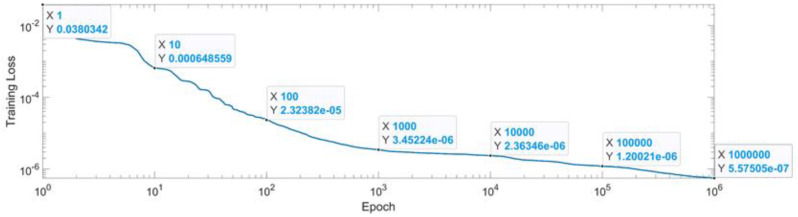
Convergence of the neural network for workspace (log scale).

Figure 31 presents the convergence of the neural network in log scale. The condition of convergence of the neural network was chosen as reaching 1 million epochs or reaching a mean squared error of $1\times 10^{-7}$. From the 1st epoch to the 1000th epoch, the MSE reached form 0.03 up to $3.4\times 10^{-6}$. From the 1000th epoch up to the 1 millionth epoch, the MSE was lowered with just one degree of magnitude. After reaching the first condition of convergence, 1 million epochs, the MSE for the last epoch is presented in Equation (42).

(42) $$MSE\_Best\_WS=5.57\times 10^{-7}$$

**Figure 32:**
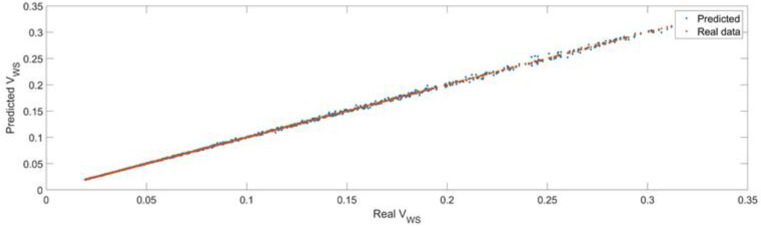
The comparison between the real data (the real volume of the workspaces) and the predicted data (the predicted values of the volume of the workspace).

Figure 32 presents a comparison between the real data, the actual values of the volume of the workspaces (validation data, evaluated numerically) and the predicted data, the value of the volume of the workspace predicted by the feedforward trained neural network. As seen in the figure, the trained model was able to predict the data with high precision.

**Figure 33:**
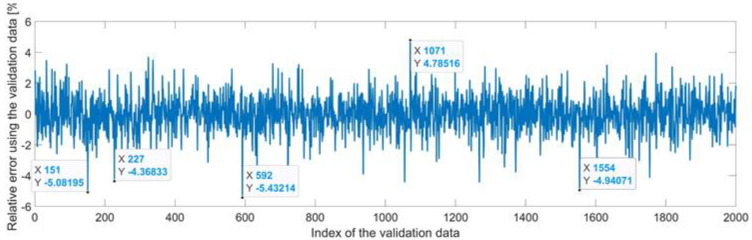
Relative errors between the target values and the predicted values for the workspace.

**Figure 34:**
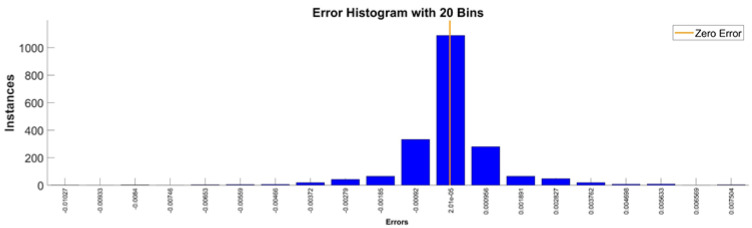
Error histogram between the target values and the estimated values for the workspace.

Figure 33 presents the relative error between the real validation data and the predicted data and the model and Figure 34 presents the error histogram between the target and predicted values.

By analyzing Figure 33 and Figure 34, the maximal error between the target and prediction was around 5%. Nevertheless, among the 2000 target values, for 327 the relative error was higher than 1%. The mean error between the target and prediction values for the first generalized coordinate was −0.0029%.

## 6. Discussion

By analyzing the numerical results from Section 4, in the case of kinematics estimation, one relevant aspect is that each generalized coordinate is estimated separately with individual neural networks for each. Therefore, the presented method may be applied even for different numbers of DOFs or type of robots. The accuracy of the trained models is provided by the MSE and the relative mean error between the real validation and estimated data, which was below 0.1%. The estimation of the kinematics using neural networks may be used in order to avoid carrying out classical kinematics algorithms for real-time implementation. Moreover, it may present advantages when applied to robots for which the kinematics problem may have more than one solution or to redundant robots. In the case of the estimation of kinematics using neural networks, the work presented may be further developed in what regards the estimation of first and second differentials of the generalized coordinates (i.e., the generalized velocities and accelerations).

In the case of estimation of the volume of the workspace, the main outcome of the proposed approach is for the process of designing or redesigning a robot. By applying this method, a human designer is able to estimate directly the volume of the workspace, without being required to numerically compute again the workspace (for each time the value of a parameters that defines the architecture of the robot has to be changed). The accuracy of the trained model is provided by the MSE and the relative mean error between the real validation and estimation data, which was below 0.1%. Still, in this case, the number of test cases that presented a relative error larger than 1% was higher than in the case of estimation of the kinematics. 

## 7. Conclusions

This paper presented the development process of an artificial neural network, namely a feedforward fully connected neural network in order to estimate the inverse kinematics and the volume of the workspace of a robot. The values of the generalized coordinates of the robot were estimated by the neural network from the poses (position and orientation) of the end effector and the volume of the workspace was estimated based on the parameters that describe the architecture of the robot. 

The datasets the neural networks were trained and validated were generated using classical inverse kinematics analysis and workspace generation for a six-DOF parallel robot composed by six identical RUS open loops that interconnect the fixed and mobile platforms. 

The topology of the neural networks was established based on two optimizations implemented in MATLAB. The optimization had as objective function the MSE and the parameters that were optimized were the number of layers, the size of each layer, the type of the activation function and the regularization rate.

In what regards the future development of this work for the estimation of the workspace, in this paper, only the volume of the workspace was considered, without implementing the shape and size of the workspace as input or output. This is why the future outlook for the estimation of the workspace is to further develop the machine learning model to be able to estimate the workspace, including the shape and dimensions. 

## Data Availability

Not applicable.
